# The Moderating Effect of Psychological Contract Violation on the Relationship between Narcissism and Outcomes: An Application of Trait Activation Theory

**DOI:** 10.3389/fpsyg.2017.01113

**Published:** 2017-06-30

**Authors:** Thomas J. Zagenczyk, Jarvis Smallfield, Kristin L. Scott, Bret Galloway, Russell L. Purvis

**Affiliations:** ^1^Department of Management, Clemson University, ClemsonSC, United States; ^2^Department of Managerial Studies, University of Illinois, ChicagoIL, United States; ^3^Sallé Galloway, Daniel IslandSC, United States

**Keywords:** narcissism, psychological contract, trait activation, turnover, neglect

## Abstract

We use trait activation and psychological contracts theories to build the argument that narcissism is a personality trait that will manifest itself in the form of exit and neglect when employees experience psychological contract violation. To test our hypotheses, we surveyed 262 employees from a wide array of industries working in different organizations at two points in time. Our results indicate that violation moderated the relationship between narcissism and exit such that narcissistic employees who experienced high levels of violation had higher levels of exit. However, we did not find support for our prediction regarding neglect. The findings suggest that the importance of narcissism at work may be contingent on the situation. Our study contributes to research on narcissism in the workplace, trait activation theory, and the role that individual differences play in shaping employee responses to psychological contract violation.

## Introduction

Narcissism – a personality construct characterized by an individual’s desire to maintain unrealistically high levels of self-esteem (Raskin et al., 1991) – has received much attention in the popular press and psychology literature in recent years. Some psychologists suggest increased narcissism in society has resulted in increased aggression and self-promotion and reduced empathy and pro-social behaviors, and is potentially even responsible for the perceived “bad behavior” of the Millennial generation (individuals born between 1982–1996; Twenge, 2006). Research exploring the effects of sub-clinical narcissism in the workplace suggests that employees with high levels of sub-clinical narcissism tend to be ambitious and highly engaged in their work (Andreassen et al., 2012) and more inclined to lead (Judge et al., 2009), but also more likely to engage in antisocial behavior (e.g., workplace incivility and counterproductive work behaviors) than employees with low levels of sub-clinical narcissism (Penney and Spector, 2002; Meier and Semmer, 2013). Narcissistic workers also tend to overestimate (relative to peers) their own leadership ability and citizenship behavior and underestimate the extent to which they engage in deviant behavior (Judge et al., 2006).

These somewhat inconsistent results highlight the need to develop greater understanding of under what circumstances narcissism is likely to result in negative employee responses at work. Indeed, if narcissism is a growing epidemic in our society – as is suggested by Twenge (2006) – then it will serve both scholars and practitioners alike to understand its implications in the workplace. Relevant to this question is the finding that individuals with high levels of narcissism tend to respond to threats negatively (Judge et al., 2006). Accordingly, we explore the relationships between narcissism and two key organization retaliatory behaviors: exit and neglect. Exit behaviors include quitting one’s job, changing jobs, and thinking of quitting or changing jobs (Hirschman, 1970). Employees engaging in neglect reduce work effort, pay less attention to quality, and demonstrate increased withdrawal in the workplace (tardiness and absenteeism; Hirschman, 1970). We reason that the characteristics of narcissists will cause them to be more likely to look for work elsewhere and withdraw from the jobs they currently have.

We explore the relationships between narcissism and exit/neglect against the backdrop of recent personality scholarship that suggests that researchers should account for the situations in which behavior occurs to better understand its effects. To do this, we draw on trait activation theory (Tett and Guterman, 2000) as an overarching theoretical framework to explain the conditions in which narcissists are most likely to engage in negative behavior at work. Trait activation theory describes how individuals’ personality traits are activated through situational circumstances and subsequently manifest themselves behaviorally. From this perspective, a situation is considered to be relevant to a trait if it provides cues for the expression of trait-relevant behavior (Tett and Guterman, 2000). In the present study, we explore narcissism in the context of employees’ responses to psychological contract violation. Employees tend to think that their organizations (as represented by supervisors and other leaders) make important promises to them regarding their jobs that are often not a part of formal written employment contracts (e.g., promises with respect to promotion, development, training, job security). Employees’ understanding of these promises form the basis of their psychological contracts, defined as schemas that include the obligations employees believe the organization has offered to them in exchange for their efforts on behalf of the organization (Rousseau, 1995). Employees often experience psychological contract violation – negative emotions such as frustration and anger (Robinson, 1996) – when they perceive that their organizations have failed to keep (i.e., have breached) their promises.

We argue that narcissism is a personality trait that will manifest itself in the form of employee exit and neglect in the face of the organization’s failure to fulfill perceived obligations to its employees. We reason that psychological contract violation will activate the negative tendencies of narcissists because such individuals tend to respond more strongly to those who threaten them by attempting to harm or derogate them (Bushman and Baumeister, 1998). In addition, the research of Penney and Spector (2002) showed that narcissism was positively related to anger, which predicted counterproductive work behavior when job constraints were high. In line with past research, we argue that the relationships that exist between narcissism and both exit and neglect will be stronger when employees experience psychological contract violation – as narcissistic employees will engage in these behaviors in an effort to harm the organization deemed to be responsible for psychological contract violation.

In sum, our research makes two contributions to the organizational literature. First, we add to the small body of research exploring the relationships between narcissism and workplace behavior by testing boundary conditions of narcissistic personality and behavior and offer a new theoretical perspective that helps to integrate individual differences into psychological contract dynamics. Second, we offer another test of trait activation theory in the workplace, which answers calls for research on the role of context in organizational behavior generally (Johns, 2006) and individual differences in the psychological contracts dynamics specifically (Bordia et al., 2008; Zagenczyk et al., 2014). Our model sheds light on the interplay of dispositional and contextual factors in predicting employee exit and neglect.

### Literature Review

Narcissism originates in Greek mythology with the story of Narcissus, a young adult who fell in love with a reflection of himself in a pool of water (Ellis, 1898). Researchers including Kernberg (1975) and Kohut (1966) argued that narcissism constitutes a personality disorder now characterized as a “pervasive pattern of grandiosity” along with a “need for admiration and lack of empathy” (APA, 2000, p. 717). However, Kohut’s (1966) research – which suggested that narcissism develops between the time an individual is an infant and when they reach adulthood - led to the development of the subclinical or ‘normal’ narcissism construct (Raskin and Hall, 1979). At a subclinical level, narcissism is derived from an attempt to regulate and maintain unrealistically high levels of self-esteem (Raskin et al., 1991). To maintain unrealistically high levels of self-esteem, narcissists tend to provide self-ratings of their own intelligence, creativity, competence, and leadership ability that are more favorable than others’ ratings of those same characteristics (John and Robins, 1994; Campbell et al., 2004; Judge et al., 2006).

As a result, narcissists have an intense desire to have their superiority reaffirmed through admiration that serves to protect and preserve their grandiose self-images (Chatterjee and Hambrick, 2006). Information that bolsters self-esteem, referred to as “narcissistic supply” (Kernberg, 1975) can be self-generated through exhibitionism or by viewing others less favorably (Bogart et al., 2004). However, some narcissistic supply must derive from the affirmation, applause, and adulation of others (Wallace and Baumeister, 2002).

The need to maintain sufficient “narcissistic supply” has important effects on the behavior of narcissists. Many of the behavioral tendencies of narcissists are an attempt to inflate and maintain their own (overly) favorable self-evaluations (Westen, 1990). As a result, narcissists engage in behaviors such as bragging (Hogan et al., 1990), derogating others (DiMaggio et al., 2002; Bogart et al., 2004), reacting to ego threats with hostility and aggression (Baumeister et al., 1996; Bushman and Baumeister, 1998; Rhodewalt and Morf, 1998; Rhodewalt et al., 1998), making internal attributions for success and external attributions for failure (John and Robins, 1994), and overestimating future outcomes and performance, even in the face of disconfirming feedback (Vazire and Funder, 2006).

Research highlights the fact that employees with higher levels of narcissism may tend to behave differently than other employees, but that these behavioral differences are most likely to occur when narcissistic supply is threatened (Bushman and Baumeister, 1998; Penney and Spector, 2002). Thus, examination of the direct relationships between narcissism and outcomes may not demonstrate the behavioral differences between employees with high vs. low levels of narcissism, but instead that the behavior may only occur in specific situations. This makes trait activation theory particularly relevant because it stems from Hattrup and Jackson’s (1996) postulation that it is critical to understand the situations in which individual differences occur in order to meaningfully comprehend how those differences affect performance in the workplace. Formally defined, trait activation is “the process by which individuals express their traits when presented with trait-relevant situational cues” (Tett and Burnett, 2003, p. 502). The basic premise of trait activation is that the degree to which a trait is likely to drive behavior is a function of the extent to which the situation provides an opportunity for or creates a necessity for the trait (Tett and Burnett, 2003). Accordingly, we suggest that it is necessary to consider the roles of both personality (e.g., narcissism) and contextual factors (e.g., psychological contract violation) in understanding the conditions under which exit and neglect are likely to occur.

Empirical research generally supports the central tenets of trait activation theory. For example, the research of Tett and Burnett (2003) revealed that the effects of personality on performance were strongest when reward contingencies were low. Similarly, Hochwarter et al. (2005) found that employees who perceived low levels of organizational support were more inclined to draw on their social skills to acquire needed resources, whereas employees who perceived high levels of organizational support were less inclined to activate their social skills – as such activation was unnecessary because resources were already provided. Kamdar and Van Dyne (2007) found the expected positive effects of conscientiousness and agreeableness on helping behaviors when social exchange relationships (e.g., leader–member exchange, team-member exchange) were negative, but not when social exchange relationships were positive. In this condition, all employees – regardless of conscientiousness and agreeableness – were more apt to perform helping behaviors because they felt obligated as a result of the favorable treatment they received from others.

In this research, we draw on psychological contracts theory to provide a situation that may activate the behavioral tendencies of employees with high levels of narcissism. Psychological contracts theory suggests that employees believe that the organization (or its agents) makes promises with respect to job security, promotion and other job conditions typically not included in written contracts. These perceived promises make up employees’ *psychological contracts*, defined as relatively stable mental models that encapsulate the perceived promises employees believe the organization has made to them in exchange for their efforts on behalf of the organization (Rousseau, 1995). Psychological contracts are important both in terms of their content, which prescribes employee attitudes and behaviors (Raja et al., 2004) as well as the degree to which they are breached (Morrison and Robinson, 1997), which usually results in reduced performance, poor attitudes and withdrawal behaviors among employees (Zhao et al., 2007). Research on outcomes of psychological contract breach is guided by social exchange theory (Blau, 1964) and the reciprocity norm (Gouldner, 1960). These theories suggest that employees form social exchange relationships with their employing organizations and respond to positive treatment by helping the organization to reach its goals and negative treatment by withholding help or actively trying to prevent the organization from succeeding (Rousseau, 1995).

A particularly important intermediate outcome of breach is psychological contract violation, defined as the “affective and emotional experience of disappointment, frustration, and anger” (i.e., psychological contract violation; Morrison and Robinson, 1997, p. 228). Psychological contract theory suggests that violation is an emotional state which follows an employee’s belief that psychological contract breach has occurred (Morrison and Robinson, 1997; Robinson and Morrison, 2000; Bordia et al., 2008) and offers a rationale for why breach results in negative attitudes and behavior. Affective events theory (AET, Weiss and Cropanzano, 1996) describes how emotions mediate the relationship between workplace events and employee responses to those events. We adopt the reasoning of Bordia et al. (2008), who suggest that psychological contract breach is an event that causes employees to experience an affective response (violation) that influences behavior and attitudes. In support of this view, Bordia et al. (2008) and Restubog et al. (2015) demonstrated a chain of events leading to workplace deviance: breach leads to violation, violation predicts revenge cognitions, and revenge cognitions predict deviant behavior. A number of other studies have shown that psychological contract violation mediates the relationship between psychological contract breach and outcomes (e.g., Turnley and Feldman, 1999; Kickul and Lester, 2001; Raja et al., 2004).

In this research, we explore the interplay of narcissism, psychological contract violation, and two dependent variables: exit and neglect. Exit and neglect are both components of Hirschman’s (1970) exit-voice-loyalty-neglect framework, which is a relatively comprehensive model for understanding employee responses to job dissatisfaction (Turnley and Feldman, 1999). Exit behaviors include movements within (e.g., changing jobs) and across organizational boundaries (e.g., quitting) as well as thinking about these movements (Hirschman, 1970). Neglect, on the other hand, occurs when an employee reduces the effort and interest he or she has in work (Vigoda, 2001). Conducting personal business on company time is an example of neglect (Rusbult et al., 1988). Exit and neglect have also been conceptualized as organizationally focused retaliatory behaviors (Skarlicki and Folger, 1997; Bennett and Robinson, 2000; Vardi and Weitz, 2004).

We generally expect that narcissism will be positively related to exit and neglect. In support of this idea, narcissists view themselves extremely favorably, partly because they have a tendency to make internal attributions for their successes (Rhodewalt and Morf, 1998) and thus tend to overrate their task performance, citizenship behavior, and leadership ability in the workplace (Judge et al., 2006). Accordingly, narcissists may feel that the treatment (in terms of compensation, promotion, developmental experiences, and benefits) is lacking for an employee of their perceived caliber. As a result, narcissists may tend to feel that another employer would recognize the knowledge, skills, and abilities that they possess which their current employer fails to recognize. They would therefore be more inclined to exit the organization, both cognitively and behaviorally (to the degree that they have options). The same arguments also lead to the prediction that employees with higher levels of narcissism will tend to engage in neglect, as they seek to harm (or at least not help) the organization in response to the insufficient treatment that they receive from the organization.

While we generally expect that employees with high levels of narcissism will tend toward exit and neglect, we argue that they will be especially inclined toward these behaviors when psychological contract violation is high. Recent psychological contracts research has positioned the organization’s failure to fulfill its promises as a threat because it reduces both tangible (e.g., Kiazad et al., 2014) and socioemotional resources (Restubog et al., 2008) of employees. Consistent with this research and the perspective of trait activation theory, we view psychological contract violation as a negative evaluation or social cue which may pose a particular threat to narcissists, as such cues threaten the preservation and protection of their desired self-image (Tesser, 1988). To this end, Kernberg (1975) demonstrated that narcissists are overly sensitive to slight insults or criticism, and they are prone to react to such situations with hostility. Based on this evidence, we suspect that when psychological contract violation occurs, narcissistic employees will be more inclined to behave in a manner which helps them to maintain their positive sense of self, which they will achieve by seeking affirmation at other organizations (through exit) and passively or actively aggressing against their own organization (through neglect).

*Hypothesis 1:* Violation will moderate the relationship between narcissism and exit. Specifically, the positive relationship between narcissism and exit will be stronger for respondents who report higher levels of psychological contract violation.
*Hypothesis 2:* Violation will moderate the relationship between narcissism and neglect. Specifically, the positive relationship between narcissism and neglect will be stronger for respondents who report higher levels of psychological contract violation.

## Materials and Methods

This study was carried out in accordance with the recommendations of the Clemson University Institutional Review Board with written informed consent from all subjects. All subjects gave written informed consent in accordance with the Declaration of Helsinki. To obtain data from full-time employees and test our hypotheses, we requested that 180 graduate and undergraduate students enrolled in organizational behavior and human resource management courses in the southern United States forward an online survey link to three full-time employees with whom they were acquainted at the beginning of the semester. Students were then requested to forward a second survey link to the same employees three months later. Students received extra credit on exams for forwarding the surveys. Employees who followed the link were given access to a secure website where they completed the survey that we developed. Responses were submitted to a secure database. The survey presented to employees at Time 1 assessed the independent variables - demographic variables, narcissism, and psychological contract violation. At Time 1, 405 employees submitted completed surveys for a response rate of 75% (405 responses/540 forwarded links). By using a student-recruited sample, we were able to obtain data from a wider range of organizations than would have otherwise been feasible, increasing our confidence in the generalizability of our findings. Although this method of sampling may result in smaller effect sizes than more traditional, non-student-recruited samples, both methods provide similar effects, and sample representativeness (Wheeler et al., 2014).

We separated the collection of our independent and dependent variables to minimize the effects of common method bias (Podsakoff and Organ, 1986). Specifically, we collected our dependent variables with a survey that was administered to the respondents three months after the initial survey was administered (Podsakoff and Organ, 1986). The second survey was administered to the respondents who completed the Time 1 survey. A total of 262 respondents completed the Time 2 surveys for a response rate of 64.7% of the Time 1 respondents and an overall response rate of 48.5%. Respondents at Time 2 were mostly male (61.8%) and Caucasian (67.6%). Mean age was 37.5 years, whereas mean organizational and job tenure were 7.03 and 4.34 years, respectively. Respondents were employed in many different white-collar positions in industries including customer service, education, and manufacturing. In terms of education, 8% of employees had high school diplomas, 9.8% had earned associates’ degrees, 42.1% had completed an undergraduate degree, and 39.3% had completed graduate school.

We used ANOVA to determine if sampling bias influenced our results. We checked for demographic differences between employees who completed Time 1 and Time 2 surveys (*n* = 262) and employees who only responded to the Time 1 survey (*n* = 405). There were no significant differences in the demographics of employees who responded to both surveys and employees who only responded to the Time 1 survey.

We used a seven-point Likert-type scale (1 = strongly disagree; 7 = strongly agree) for all items except narcissism and the control variables. Items were coded so that a higher score indicated a higher level of the focal construct (with the exception of reverse-coded items). We averaged employee responses to the items associated with a particular scale to construct the measures that were used.

We measured subclinical narcissism using a 16-item (NPI-16) scale developed by Ames et al. (2006). The NPI-16, which is a shorter subset of items from the NPI-40 (Raskin and Terry, 1988), is recommended for field studies and other situations in which respondents may be unwilling or unable to fill out the NPI-40 (Ames et al., 2006). The NPI-16 has psychometric properties similar to those of the NPI-40 (Ames et al., 2006). For each of the 16 questions item, there is a narcissistic response and a non-narcissistic response. A sample item requires respondents to choose between two choices: “I am an extraordinary person” and “I am much like everybody else”. Cronbach’s alpha for the scale was 0.79.

To assess psychological contract violation, respondents completed Robinson and Morrison’s (2000) four item psychological contract violation measure at Time 1. Cronbach’s alpha for the scale was.95. A sample item is, “I feel a great deal of anger towards my organization”.

We measured exit or turnover intentions with Bluedorn’s (1982) three-item scale. A sample item is, “I am seriously thinking about quitting my job”. Cronbach’s alpha for the scale was 0.87.

Neglect was measured using three items from Rusbult et al. (1988) scale. The three items included were “Now and then there are workdays where I just don’t put much effort into my work”, “I have quit caring about my job and will allow conditions to get worse and worse”, and “I feel like putting less effort into my work”. Cronbach’s alpha for the scale was 0.72. This is in line with the reported reliabilities of the full, six-item neglect scale from Rusbult et al. (1988), which ranged between 0.69 and 0.82.

Based on previous research, we controlled for a number of variables; gender (1 = male and 2 = female), age (years), tenure (years), and race (1 = Caucasian, 2 = African-American, 3 = Asian, 4 = Hispanic, and 5 = other). We measured tenure because it is negatively related to exit (Mobley et al., 1978). Similarly, age (measured in years) may also have a negative relationship with exit (Ng and Feldman, 2009). Further, we measured gender (male = 1, female = 2) because females tend to have higher exit behaviors than males (Weisenberg and Kirschenbaum, 1993). Employees who are older and have greater tenure are less likely to engage in neglect behaviors (Ng and Feldman, 2008), so we controlled for these in our tests of the relationship between narcissism and neglect. Finally, a number of studies demonstrate that females are less likely to engage in counterproductive workplace behaviors than males (Hershcovis et al., 2007), further indicating the need to control for gender.

## Results

**Table 1** includes the descriptive statistics and inter-correlations for all of the variables included in the study. **Table 2** includes the results of moderated regression analysis used to test hypothesis 1, whereas **Table 3** presents the results of our test for hypothesis 2. We mean-centered the independent variable (before creating the interaction term; Aiken and West, 1991). We included the control variables in the first step of the regression, our independent and moderator variables in the second step, and our interaction term in the third step.

**Table 1:** Descriptive statistics and zero-order correlations of the study variables.

Variables	*M*	*SD*	1	2	3	4	5	6	7	8
1. Age	37.47	13.10								
2. Sex	–	–	0.09							
3. Race	–	–	***–0.29***	**–0.13**						
4. Tenure	7.03	8.71	***0.64***	0.03	*–0.****22***					
5. Narcissism	1.59	0.24	***0.16***	–0.02	–0.04	***0.12***	(0.79)			
6. Violation	2.29	1.48	***–0.20***	–0.01	**0.13**	–0.01	-0.01	(0.95)		
7. Exit	2.78	1.68	***–0.30***	–0.05	0.11	0.05	-0.10	***0.49***	(0.87)	
8. Neglect	2.63	1.26	***–0.37***	**–0.12**	***0.19***	***–0.17***	-0.07	***0.32***	***0.53***	(0.72)

*N = 262. Bold correlations are significant at *p* < 0.05. Bold and italic correlations are significant at *p* < 0.01.*

**Table 2:** Hierarchical moderated regression predicting T2 Exit from T1 narcissism and T1 psychological contract violation.

		β					
Variable and step	Step 1	Step 2	Final	*R^2^*	*ΔR^2^*
Step 1: Controls				0.08^∗∗^	0.08^∗∗^
Age	–0.02	–0.01	–0.01		
Sex	0.01	0.00	0.01		
Race	–0.28^∗∗^	–0.24^∗∗^	–0.24^∗∗^		
Tenure	0.03	0.01	0.02		
Step 2: Main effects				0.28^∗∗^	0.20^∗∗^
Narcissism		–0.35^∗∗^	–0.35^∗∗^		
Violation		–0.18^∗∗^	–0.18^∗∗^		
Final Step: Interaction terms (centerd)				0.30^∗∗^	0.02^∗∗^
Narcissism × Violation			–0.13^∗^		

*N = 262. Standardized coefficients are reported. ^∗^*p* ≤ 0.05; ^∗∗^*p* ≤ 0.01.*

**Table 3:** Hierarchical moderated regression predicting T2 neglect from T1 narcissism and T1 psychological contract violation.

		β					
Variable and step	Step 1	Step 2	Final	*R^2^*	*ΔR^2^*
Step 1: Controls				0.16^∗∗^	0.16^∗∗^
Age	–0.39^∗∗^	–0.33^∗∗^	–0.33^∗∗^		
Sex	–0.08	–0.08	–0.08		
Race	0.10	0.09	0.09		
Tenure	0.08	0.07	0.07		
Step 2: Main effects				0.22^∗∗^	0.06^∗∗^
Narcissism		–0.03	–0.02		
Violation		0.24^∗∗^	0.24^∗∗^		
Final Step: Interaction terms (centerd)				0.22^∗∗^	0.00
Narcissism × Violation			–0.05		

*N = 262. Standardized coefficients are reported. ^∗^*p* ≤ 0.05; ^∗∗^*p* ≤ 0.01.*

**FIGURE 1:**
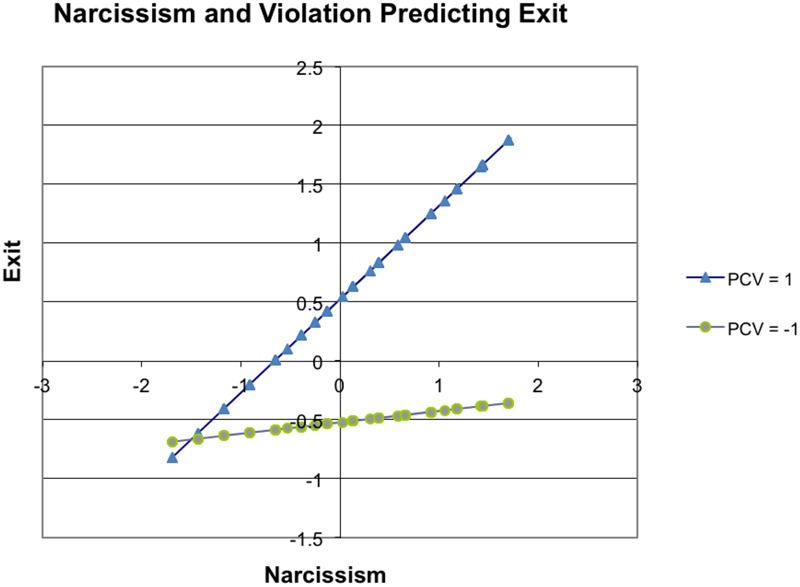
Interaction between narcissism and psychological contract violation predicting exit.

Hypothesis 1 predicted that violation would moderate the relationship between narcissism and exit. This hypothesis was supported (see **Table 2**). The interaction term (narcissism × violation) was significantly associated with turnover intentions (β = –0.13, *p* ≤ 0.05), after accounting for control variables and main effects. Inclusion of the interaction term resulted in the explanation of a significant amount of variance in predicting exit at Time 2 (Δ*R*^2^ = 0.02, *p* < 0.05), after accounting for control and main variables. A significant simple slope emerged only for employees with high levels of violation (*t* = 3.74, *p* < 0.001), not for employees with low levels of violation (*t* = 0.457, *p* = 0.678). The narcissism-violation interaction suggests that narcissism had a stronger positive relationship with turnover intentions for respondents with high levels of violation compared to respondents with low levels of violation (see **Figure 1**). Interestingly, we found a significant and positive association between race and exit in our analysis. We explored this further using ANOVA and found that Asian employees (mean = 3.10) had a significantly higher level of exit relative to American (mean = 2.67) employees (*F* = 3.95, *p* < 0.05, df = 1,267).

Hypothesis 2, which predicted that violation would moderate the relationship between narcissism and neglect, was not supported (see **Table 3**). The interaction term (narcissism x violation) was not significantly associated with neglect (β = –0.05, n.s.).

## Discussion

Results of our study of 262 employees from various occupations demonstrated that the relationship between narcissism and exit is stronger when employees report high as opposed to low levels of psychological contract violation. However, we did not find evidence that violation moderated the relationship between narcissism and neglect. Accordingly, our research makes a number of contributions to the literature. First and foremost, our study contributes to the relative dearth of research on the consequences of narcissism in the workplace. In the seminal publication on narcissism in the I–O psych/management literature, Judge et al. (2006) reported that 0 articles had been published in top I–O psychology journals. This number is surprising in light of two factors: (1) the intense interest that both management and I–O psychology scholars have in counterproductive and deviant behaviors at work, and (2) the potential that narcissism has – based on reviews in the psychology literature (e.g., Bushman and Baumeister, 1998) – to predict such behaviors. Given this, one might wonder why so few studies have explored narcissism as an individual difference variable that can help scholars and managers predict bad behavior in the workplace. The insignificant results that we obtained when direct relationships between narcissism and our dependent variables (exit and neglect) point to one potential reason why there may be so few published studies: it may be difficult to establish direct relationships between narcissism and outcome variables.

The reasoning above highlights the second contribution of our work, which is the advancement of trait activation theory as a theoretical framework through which we can – hopefully - understand the effects of narcissism in the workplace. Much of the psychological research on narcissism highlights the fact that narcissists tend to “lash out” against others in response to situations in which their self-esteem is threatened (e.g., Bushman and Baumeister, 1998). This suggests that, in routine situations, we may not witness the effects of narcissism at work, but instead that this may largely be observed when threat occurs. Our results suggest that psychological contract violation is a situation which is likely to “activate” dispositional characteristics within employees which are potentially harmful to the organization – at least when exit is considered as a dependent variable. Notably, our research is in line with past research on trait activation theory, which has utilized social exchange variables such as perceived organizational support as situational factors expected to interact with employee personality in predicting performance at work (Hochwarter et al., 2005; Kamdar and Van Dyne, 2007). Therefore, in our estimation, trait activation is a particularly appealing theoretical framework for future research exploring the effects of narcissism in the workplace because it integrates situational factors that may be important to understanding the effects of narcissism.

Third, our work contributes to understanding the role of personality in psychological contract dynamics. Extant research in this area indicates that personality variables significantly affect the manner in which employees respond to psychological contract breach. For example, variables such as equity sensitivity (Kickul and Lester, 2001; Restubog et al., 2009), conscientiousness (Orvis et al., 2008), self-control (Bordia et al., 2008; Restubog et al., 2015), and Machiavellianism (Zagenczyk et al., 2013) moderate the relationship between breach and outcomes. However, with the exception of the work of Zagenczyk et al. (2013), research has largely neglected the role of “dark” personality traits in the psychological contract dynamics, despite the fact that employees with dark traits may be more inclined to retaliate in response to perceived negative treatment (e.g., Meyer, 1992). This may emerge as a more important issue as organizations and researchers become more aware of the costs stemming from unfulfilled psychological contracts, particularly among leaders, who tend to have higher levels of Machiavellianism and narcissism than do non-leaders (Judge et al., 2009).

In particular, we are among the first researchers to explore the role that personality plays in the context of psychological contract violation, a variable that receives relatively little attention compared to the more commonly studied psychological contract breach variable. Recently, scholars have called for research exploring the role of psychological contract violation in employer-employee relationship research (Dulac et al., 2008). Notably, most past research on psychological contract violation has examined its utility as a mediator of the relationship between psychological contract breach and outcomes – that is, breach (the employee’s cognition that the organization has failed to fulfill its obligation to him/her; Morrison and Robinson, 1997) results in negative emotions which then lead to negative attitudes and behaviors on the part of employees. Recent research by Bordia et al. (2008) and Restubog et al. (2015) has highlighted the fact that breach results in violation, which causes employees to seek revenge against the organization. Although our study did not explore revenge cognitions directly, it is possible (and, given recent research in psychology, perhaps likely) that the experience of psychological contract violation by narcissistic employees triggers the need to take revenge.

To this end, it bears mentioning that exit is regarded as an active response to dissatisfying work conditions, whereas neglect is commonly considered to be a more passive response (Rusbult et al., 1988). In light of this reasoning, the fact that we found evidence for the moderating effect of violation on the narcissism-exit relationship – but not the narcissism-neglect relationship – makes sense, given that most examinations of narcissism in the psychology literature have explored its relationship with aggressive (as opposed to passive-aggressive) behaviors (Kernberg, 1975; Bushman and Baumeister, 1998).

Another potential explanation for the absence of increased neglect might be related to narcissistic supply. Neglect could easily result in immediate negative feedback and a corresponding reduction in external affirmation. A specific psychological contract breach does not necessarily diminish the short-term level of narcissistic supply that an individual gets from the more general work environment. In fact, the precipitating psychological contract breach that leads to an exit intention might even create the need for an individual to increase narcissistic supply at work through other colleagues. For example, recent research by Andreassen et al. (2012) underscored the high level of involvement that narcissists have in their work. Thus, if nothing else, continued diligence in the work environment would serve to support the narcissistic tendency to self-affirm.

Our work has practical implications as well. Employers must be especially sensitive to employees with high levels of narcissism when psychological contract violation is likely to occur, as these employees may be more apt to seek new employment in such situations. This result is particularly relevant when one considers that the tendencies of narcissistic employees, such as a strong need for achievement, facilitates their emergence as leaders (e.g., Deluga, 2001; Judge et al., 2009). On a somewhat related note, there may be value to measures of narcissism (such as the NPI) that may eventually serve as important selection tools, particularly when recruiting for leadership positions. In particular, research by Twenge (2006) suggests that college graduates are becoming increasingly narcissistic with each passing year. Much has been made of “the Millennials” or Generation Y in the popular press and organizational literature of late (for a review, see Deal et al., 2010). If in fact narcissism is on the rise to the same degree in the general population as it is in Twenge’s samples of college graduates, the workplace could indeed become a more hostile and aggressive place in the future. In such a scenario, organizational success may be more heavily dependent on reducing the frequency of psychological contract violation or mitigating its effects when it occurs.

Our study has some limitations that need to be mentioned as well. First, it is possible that employees with high and low narcissism varied with respect to exit and neglect at Time 1, as we did not measure these variables in our initial wave of data collection. Future research can provide causal evidence for the ordering of our variables through longitudinal research or experimental designs. Second, we obtained all of our data from employee self-reports, so common method bias is a concern. We separated collection of our independent and dependent variables temporally to reduce this effect (Podsakoff and Organ, 1986). Future research that explores other-rated (supervisor- or coworker-rated citizenship, deviance, and performance) or objective measures (turnover) could extend our research further.

## Conclusion

Despite the scholarly attention paid to the construct of narcissism, there is a relative lack of research into the organizational consequences of this personality trait. In this study, we focus on the interaction of employee narcissism with the circumstance of psychological contract violation to better understand how this personality trait leads to outcomes. Our finding that narcissism more strongly predicts exit in the presence of psychological contract violation indicates that scholars should pay increasing attention to how contextual situations may cause or alter the relationship between narcissism and organizationally important outcomes.

## Author Contributions

TZ, data analysis and primary author; JS, conducted data analysis and aided in data collection; KS, data collection, analysis, and writing; BG, data collection and writing; RP, data collection and analysis.

## Conflict of Interest Statement

The authors declare that the research was conducted in the absence of any commercial or financial relationships that could be construed as a potential conflict of interest.
